# Age estimation and growth patterns in young harbor seals (*Phoca vitulina vitulina*) during rehabilitation

**DOI:** 10.1093/jmammal/gyae128

**Published:** 2024-12-10

**Authors:** Beatriz Rapado-Tamarit, Margarita Méndez-Aróstegui, Koen de Reus, Tom Sarraude, Ido Pen, Ton G G Groothuis

**Affiliations:** Behavioural Biology, Groningen Institute for Evolutionary Life Sciences, University of Groningen, 9700 CC Groningen, The Netherlands; Sealcentre Pieterburen, Hoofdstraat 94A, 9968 AG Pieterburen, The Netherlands; Behavioural Biology, Groningen Institute for Evolutionary Life Sciences, University of Groningen, 9700 CC Groningen, The Netherlands; Sealcentre Pieterburen, Hoofdstraat 94A, 9968 AG Pieterburen, The Netherlands; Artificial Intelligence Lab, Vrije Universiteit Brussel, 1050 Brussels, Belgium; Comparative Bioacoustics Group, Max Planck Institute for Psycholinguistics, 6525 XD Nijmegen, The Netherlands; Donders Institute for Brain, Cognition and Behaviour, Radboud University, 6500 GL Nijmegen, The Netherlands; Behavioural Biology, Groningen Institute for Evolutionary Life Sciences, University of Groningen, 9700 CC Groningen, The Netherlands; Department of Biology, University of Turku, FI-20014 Turku, Finland; Theoretical Research in Evolutionary Life Sciences, Groningen Institute for Evolutionary Life Sciences, University of Groningen, 9700 CC Groningen, The Netherlands; Behavioural Biology, Groningen Institute for Evolutionary Life Sciences, University of Groningen, 9700 CC Groningen, The Netherlands

**Keywords:** age estimation, canine length, compensatory growth, dorsal standard length, growth patterns, Harbor Seal, morphometric measurements, *Phoca vitulina vitulina*, crecimiento compensatorio, estimación de la edad, longitud del canino, longitud dorsal estándar, medidas morfométricas, patrones de crecimiento, *Phoca vitulina vitulina*

## Abstract

To study patterns in behavior, fitness, and population dynamics, estimating the age of the individuals is often a necessity. Specifically, age estimation of young animals is very important for animal rehabilitation centers because it may determine if the animal should be taken in and, if so, what care is optimal for its rehabilitation. Accurate age estimation is also important to determine the growth pattern of an individual, and it is needed to correctly interpret the influence of early body condition on its growth trajectories. The purpose of our study was to find body measurements that function as good age estimators in young (up to 3 months old) harbor seals (*Phoca vitulina vitulina*), placing emphasis on noninvasive techniques that can be used in the field. To meet this goal, body mass (BM), dorsal standard length (DSL), upper canine length (CL), body condition (BC), and sex were determined from 45 Harbor Seal pups of known age. Generalized additive mixed models were fitted to find how well these morphometric measures predicted age, and the results from the selected model were used to compute growth curves and to create a practical table to determine the age of young animals in the field. We found that both DSL and CL—and to some extent sex—were useful predictors for estimating age in young harbor seals and that the growth rate of pups raised in captivity is significantly lower than for those raised in the wild. In addition, we found no evidence for compensatory growth, given that animals that arrived at the center with a poor BM or BC continued to show lower BM or BC throughout almost the entire rehabilitation period.

## Teaser

Study on young harbor seals reveals that body measurements such as body and teeth length can accurately estimate their age, aiding in conservation and rehabilitation efforts. Captive-raised pups grow differently from those in the wild, and initial poor body condition persists during rehabilitation.

## Resumen

Para estudiar patrones de comportamiento, eficacia biológica y dinámica poblacional, a menudo es necesario estimar la edad de los individuos. Específicamente, la estimación de la edad de animales jóvenes es muy importante para los centros de rehabilitación, ya que puede determinar si el animal joven debe ser ingresado y, en caso afirmativo, qué cuidados específicos serían los óptimos para su rehabilitación. La estimación precisa de la edad también es importante para determinar el patrón de crecimiento de un individuo, y es necesaria para interpretar correctamente la influencia de la condición corporal temprana en sus trayectorias de crecimiento. El propósito de nuestro estudio era encontrar medidas corporales que funcionen como buenos estimadores de edad en focas comunes jóvenes (de hasta 3 meses de edad; *Phoca vitulina vitulina*), poniendo énfasis en técnicas no invasivas que puedan usarse en el campo. Para alcanzar este objetivo, se ha medido la masa corporal (BM), la longitud dorsal estándar (DSL), la longitud del canino superior (CL), la condición corporal (BC) y el sexo de 45 crías de foca común de edad conocida. Se ha aplicado modelado estadístico para determinar la eficacia de las medidas morfométricas para predecir la edad, y los resultados del modelo seleccionado se han usado para calcular las curvas de crecimiento y crear una tabla práctica para usar en el campo. Descubrimos, por un lado, que tanto DSL como CL, y hasta cierto punto el sexo, parecen ser predictores importantes para estimar la edad en focas comunes jóvenes, y, por otro, que la tasa de crecimiento de las crías alimentadas en cautividad es significativamente menor que la de las criadas en la naturaleza. Además, no encontramos evidencia de crecimiento compensatorio, dado que los animales que llegaron al centro con una mala masa o condición corporal continuaron mostrando una masa o condición corporal más baja durante casi todo el período de rehabilitación.

Being able to accurately estimate the age of individuals is important because age is often key to studies pertaining to animal behavior, fitness, and population dynamics. Moreover, age estimation of mammals is especially important for animal rehabilitation centers because it may determine if an animal should be taken in and, if so, what specific care is optimal for its rehabilitation. Knowing the correct age not only provides insights into population structure and expected growth patterns but also recognizes the age-dependent relationship between food and growth. Additionally, having accurate age information facilitates the assessment of whether animals arriving in a relatively poor state demonstrate accelerated growth during rehabilitation, also known as compensatory growth (Metcalfe and Monaghan 2001). Compensatory growth can help an organism catch up after a period of slowed growth, but may reduce the quality of its physical traits, emphasizing the importance of considering other strategies such as allowing more time to reach normal body mass.

Historically, Atlantic harbor seals (*Phoca vitulina vitulina*), called harbor seals hereafter, have been rehabilitated in the Netherlands since the 1950s when the first rehabilitation center was built on the island of Texel. Rehabilitation efforts aimed to fight the decrease in population numbers due to hunting and outbreaks of the phocine distemper virus in 1988 and 2002 (Brasseur 2018). Despite the Wadden Sea housing the largest harbor seal population in Europe with approximately 33,300 individuals (Common Wadden Sea Secretariat 2022; Galatius et al. 2023), the practice rehabilitating harbor seals is prevalent not only in the Netherlands but also in other countries around the Wadden and North Sea, including Germany and the United Kingdom. These rehabilitation centers primarily care for 2 groups of patients: young harbor seals up to 3 months old and older seals that are injured or sick, often with parasitic pneumonia.

The age of seals is usually estimated by counting the annual growth rings laid down in the cementum of their teeth (Lockyer et al. 2010). This method is an invasive technique that requires putting the animal under anesthesia, and it estimates the age of an individual with a resolution of only 1 year. Thus, this technique has limited use for a more refined estimation or for animals younger than 1 year old. Other less invasive techniques include analysis of the pelage condition and the use of body measurements. Unfortunately, analysis of the pelage can only differentiate individuals between animals younger or older than 1 year (Corpe et al. 1998) due to the different qualities of the hairs. Body mass in pups varies based on the quality and quantity of nursing received, the timing of weaning, and how long it takes them to learn how to hunt (Muelbert and Bowen 1993). The condition of the umbilical cord can be used to age pups to some extent, but as the cord drops off between Day 6 and 10 (Dierauf et al. 1986), its usefulness for older than 10 days of age estimation is nonexistent. Weaning happens quite abruptly, around 21 to 24 days old (Muelbert and Bowen 1993; Cordes and Thompson 2013), but can sometimes take up to 4 weeks. During the first 5 weeks following weaning, weaners lose about 21% of their body mass while they learn how to hunt (Muelbert and Bowen 1993). Thus, body mass alone cannot be used as a measure to age young weaners. Rehabilitation centers need to determine if a seemingly stranded young seal is a nursing or weaned pup. A nursing pup would be brought into rehabilitation if it is in poor body condition and has lost weight in the last 24 h; however, a weaned pup should not go through rehabilitation, even when underweight (according to the regulations of the Ministery of Agriculture, Nature and Food Quality of the Netherlands 2020).

The purpose of our study was to find body measurements that can be used for constructing good age estimators in young harbor seals, placing emphasis on noninvasive techniques that can be used in the field. This study will help make more informed decisions prior to taking animals into rehabilitation and will allow observation of their growth patterns in captivity. To meet this goal, body mass (BM), dorsal standard length (DSL), upper canine length (CL), axillary girth (AG), and body condition (BC) were taken from 45 harbor seal pups of known age. These seals were brought into rehabilitation at Sealcentre Pieterburen during the spring and summer of 2018, and age was determined at arrival by veterinarians based on the state of the umbilical cord. Statistical modeling was performed to test how well morphometric measures predicted age. Results from the selected model were used to compute growth curves and to create a practical table for usage in the field. Growth patterns were then statistically compared between pups that arrived with poor or good BM and BC, assessing the presence or absence of compensatory growth—additionally, they were compared with growth patterns extracted from the literature of wild harbor seals to evaluate the efficiency of captive feeding protocols.

## Materials and methods

### Study area

The study was conducted in Sealcentre Pieterburen, a rehabilitation center for sick, injured, and orphaned seals in the Netherlands during the spring and summer of 2018. The subjects of the present study are Harbor Seal pups that were found along the mainland coast of the Netherlands and the Dutch Wadden Sea islands (except Texel; Supplementary Data SD1). They were brought to the Sealcentre for rehabilitation because they were considered orphaned, having lost at least 300 g in the last 24 h and with no females in the vicinity (Wilson 2009). The weight of an animal was initially measured in the field using a digital luggage scale and a weighing sling. Twenty-four hours later, they were reexamined, though they were not continuously monitored during this interval. The absence of females nearby was inferred from the lack of tracking marks in the mud around the pups.

### Measurements

**Fig. 1.**
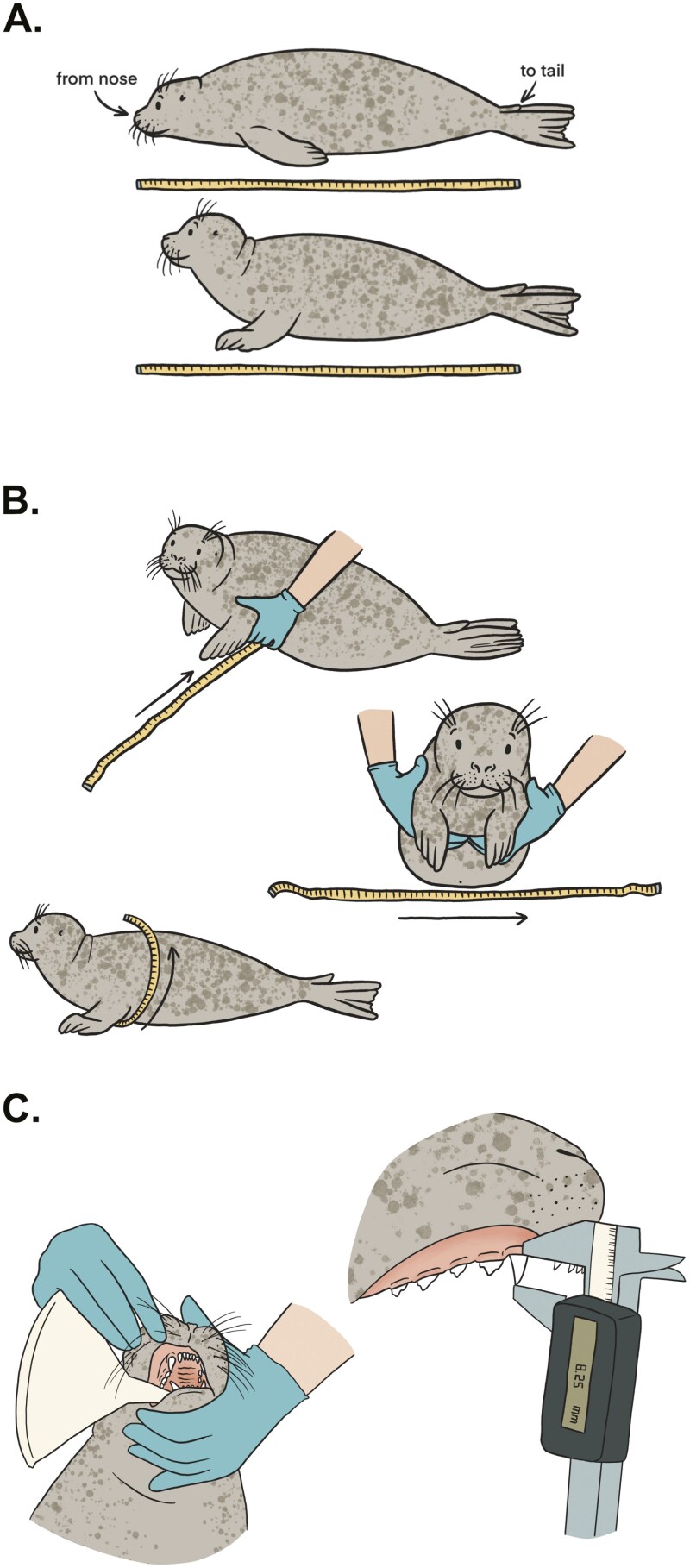
Measurement techniques. Panel A shows how to measure DSL. The top image shows the animal in a restrained position, whereas in the bottom image it is shown in the restrained but more natural position. Panel B shows how to measure AG and Panel C how to measure CL.

Sex, BM, DSL, AG, CL (of the upper right canine), and fat score were measured for all pups that arrived at the Sealcentre between 20 May up until 8 July of 2018. Pups were sexed by checking the perianal region; females present 2 openings (anus and vagina) between the hind flippers, and males present only the anus. To measure BM, pups were placed in a wicker basket and put on a digital scale (Universal Weight Electronics, Model No.: AFS-150) that can measure with an accuracy of 0.05 kg. The weight of the basket was later subtracted. To measure DSL, AG, and CL pups were restrained on their ventral area by professional caretakers from Sealcentre Pieterburen; a towel was always used to cover the eyes of the animals to minimize their stress. DSL, defined as the straight line from the tip of the nose to the end of the tail (McLaren 1993), was measured by the veterinarian and the first author (BR-T) using a measuring tape to the nearest centimeter (Fig. 1A). To get a standard measurement of DSL, the caretaker restrained the head of the animal against the floor and aligned the zero mark of the measuring tape with the tip of the nose. The caretaker would then immediately hold the pup in a more natural position with its head up to minimize discomfort. AG was measured behind the axilla of the front flippers of the pup to the nearest centimeter (Fig. 1B)—the chest was gently lifted by the caretaker so that the measuring tape could be tightly strapped around it. CL was measured with a digital caliper (Powerfix Profi, IAN 291710) with an accuracy of 0.01 mm (Fig. 1C). The caretaker kept the mouth of the pup open placing the narrow end of a plastic funnel between the upper and lower jaw, between the second and third postcanine—the fixed upper jaw of the caliper was placed at the border of the gums with the protruding canine and the movable lower jaw at the tip of the canine. For each animal, after the initial measurements taken at arrival, BM, DSL, AG, and CL were taken once a week. To avoid disturbing them unnecessarily, the sampling was always done on the same day that the pups were scheduled to be weighed for veterinary purposes. Measurements were taken until release or death of the animal. At arrival, the fat score of all pups was determined by veterinarians based on the estimated fat deposition in the pelvic and neck region of the animal, following the guidelines of Merck (2013; Supplementary Data SD2). Furthermore, the BC of all individuals was calculated using both AG and DSL.

Data were collected for all animals that arrived at the Sealcentre, but only data from pups younger than 10 days old at arrival were used. This age window was taken to ensure that age could be reliably estimated based on the status of the umbilical cord (Dierauf et al. 1986), compared to older pups which arrive without an umbilical cord or with the belly button fully healed. This reduced the data set to 50 individuals. Another 4 individuals were removed from this reduced data set before performing the analysis because there were not enough data: 2 died within 5 days after arrival; 1 was euthanized shortly after entering the rehabilitation process due to the appearance of severe neurological signs; and another was euthanized 14 days after arrival due to lack of improvement despite treatment for its fever, lethargy, and digestive system problems. Upon examination of the data, an additional animal (seal 18-081) was excluded from the analysis due to its outlier status. This individual exhibited no dental growth during the initial 2-week period, a phenomenon deemed exceptional and not previously documented among Harbor Seal pups at the Sealcentre (Results section). This resulted in 428 observations from 45 individuals, staying at the Sealcentre for a period ranging from 45 to 94 days—all had fat scores ranging from 1 (emaciated) to 4 (underweight), indicating overall poor condition.

### Ethical statement

No invasive sampling was performed, and no special permit was needed. Admission and rehabilitation procedures of harbor seals at the Sealcentre Pieterburen were authorized by the government of the Netherlands (permission ID at time of sample-taking: FF/75/2012/015).

### Statistical analysis

Statistical analyses were performed using R statistical software (v4.4.0 alpha; R Core Team 2024) assisted by the integrated development environment RStudio (v2023.12.1; Posit Team 2024). We: (i) examined the interrater reliability of our body measurements; (ii) fitted and compared several models to estimate the age of pups in captivity; (iii) analyzed their growth curves; (iv) compared our data with animals raised in the wild; and (v) tested for the presence of compensatory growth.

#### Interrater reliability

Since DSL and AG were measured twice, once by the veterinarian on duty and once by BR-T, the intraclass correlation coefficient (ICC) was used to test the interrater reliability (Stemler 2004; Liu et al. 2016). We used the *icc* function from the *irr* R package (v0.84.1; Gamer et al. 2019). We specified the type of model as 2-way because both subjects and raters are viewed as random effects. We computed the agreement based on the single value of each rater. For the different pairs of researchers, the ICC for DSL and AG ranges between 0.78 and 0.90, and 0.91 and 0.99, respectively. Since for both measurements, the ICC was higher than 0.75, they were considered sufficiently reliable across raters (Koo and Li 2016). For each measurement, we used the average value of both raters for statistical analyses.

#### Predicting the age of our study subjects

When pups arrived at the Sealcentre, their age was estimated by on-site veterinarians using the established criteria from Dierauf et al. (1986; Supplementary Data SD3). Subsequently, age was calculated by adding on the difference in the number of days between the measurement date and the arrival date to the age estimated at arrival. We modeled the data using the estimated age as the response variable and several morphometric measurements (i.e., DSL, CL) and sex of the animal as predictors.

Our aim was to determine to what extent the abovementioned morphometric measurements could predict the age of pups and young weaners between 0 to 9 weeks old (given that they are one of the main patient groups in the rehabilitation centers). However, to ensure a more accurate model fit, we used all data that we collected until the animals were released (up to a maximum of 16 weeks). BM and AG were not included in the model because they are highly dependent on the nursing performance of the mother and when, during the lactation period, a pup is found (Skinner 2006). Therefore, they are considered unreliable predictors for estimating the age of stranded pups. For this reason, only DSL, CL, and sex were included. Due to the nonlinear growth curves of DSL and CL (Supplementary Data SD4 and SD5) and the presence of repeated measurements, we chose to employ generalized additive mixed models (GAMMs) for analysis, with all models fitted using the *mgcv* package (v1.9-1; Wood 2017). We assessed model residuals to verify adherence to distributional assumptions, always assuming a Gaussian error distribution, which was validated through Q–Q plot inspections. Additionally, we employed an identity link and the default thin plate spline smoothers in all models. Our approach involved a stepwise background elimination regression to identify the most suitable model for predicting new data. To initiate our model selection process, we started by constructing the most comprehensive model possible. This model incorporated DSL, CL, their interaction, as well as sex and the interaction of sex with DSL and CL, all as fixed effects. To establish the interaction between DSL and CL, we employed the ti() function. Additionally, we introduced sex-specific smoothers to capture each potential interaction between sex and DSL/CL. Furthermore, to address the issue of repeated measurements and to capture individual “random” variation in the shape of growth curves, we incorporated random factor smooths, using the bs=“fs” argument in the smoothing functions.

The GAMMs were fitted using a restricted maximum likelihood (REML) approach, except when we compared models that differed in their fixed effects, in which case they were fitted using maximum likelihood (Wood 2011). We compared all models and ranked them using the adjusted Akaike information criterion (AICc; Burnham and Anderson 1998). The model with the lowest AICc was selected as it was deemed to have the best predictive ability for new data, henceforth referred to as age model.

#### Creating a practical guideline

**Fig. 2.**
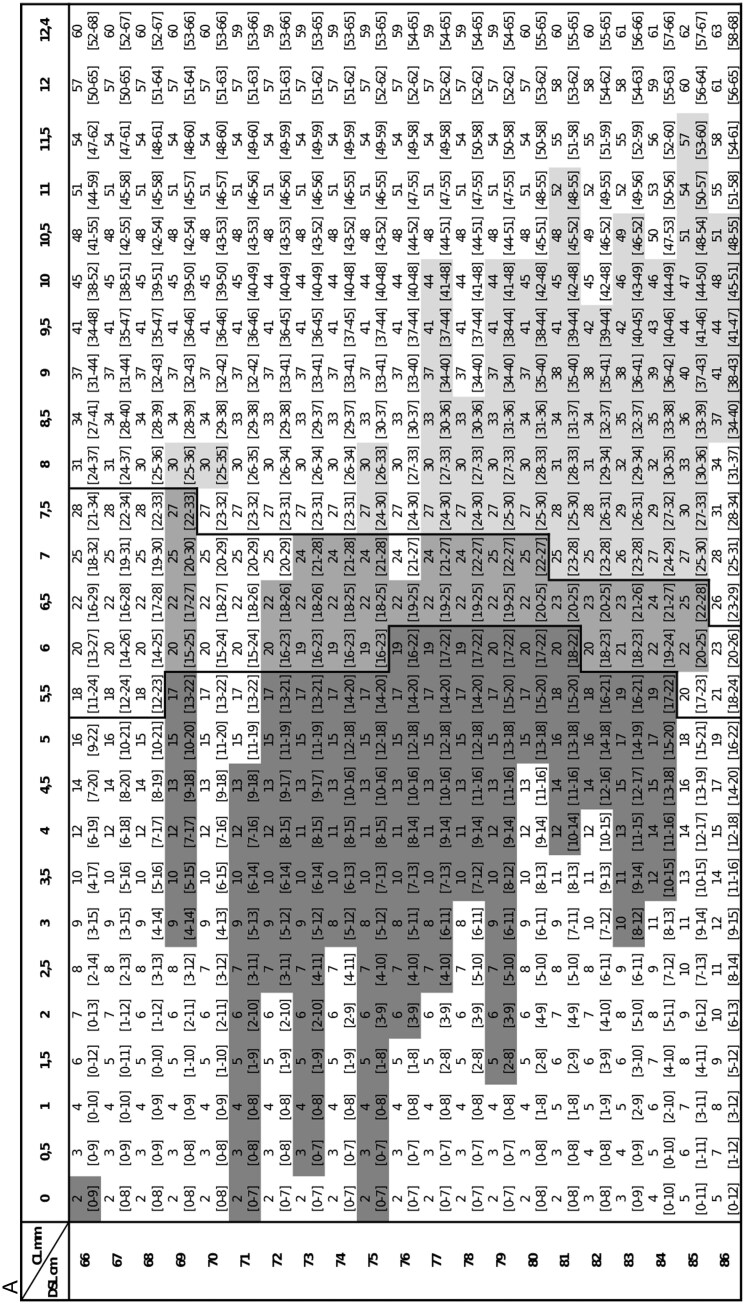

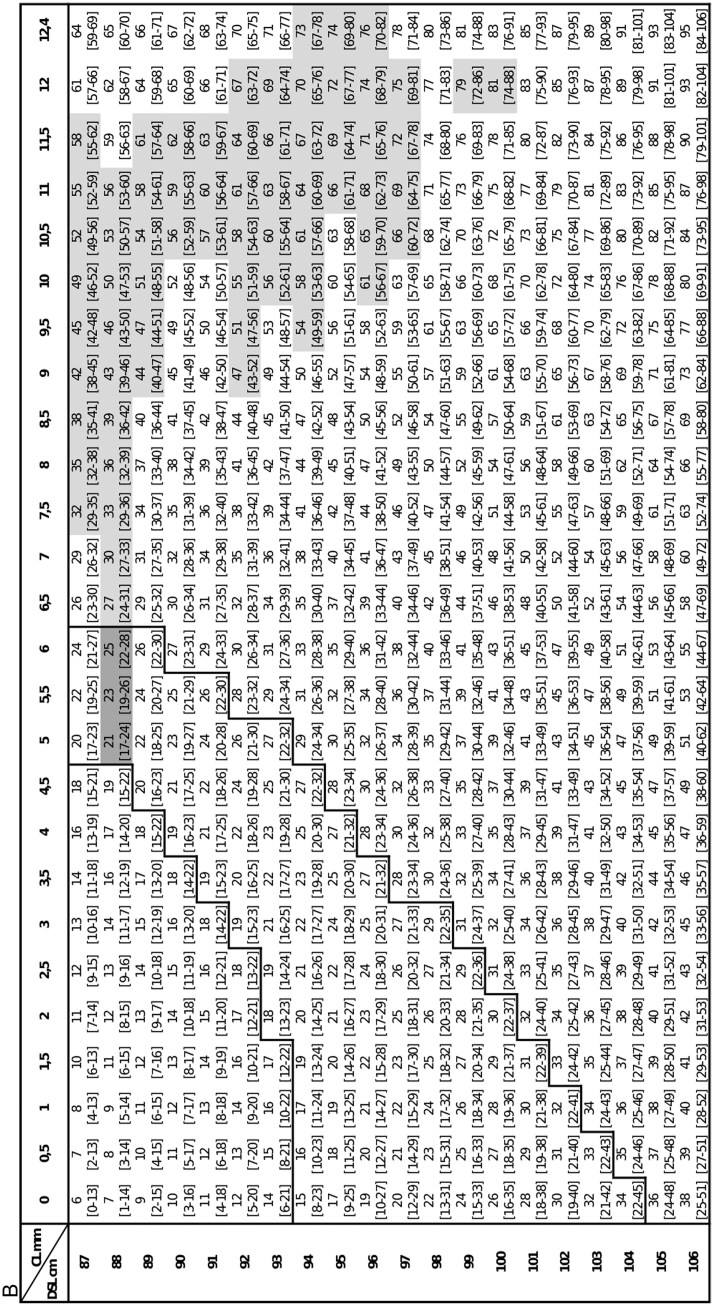

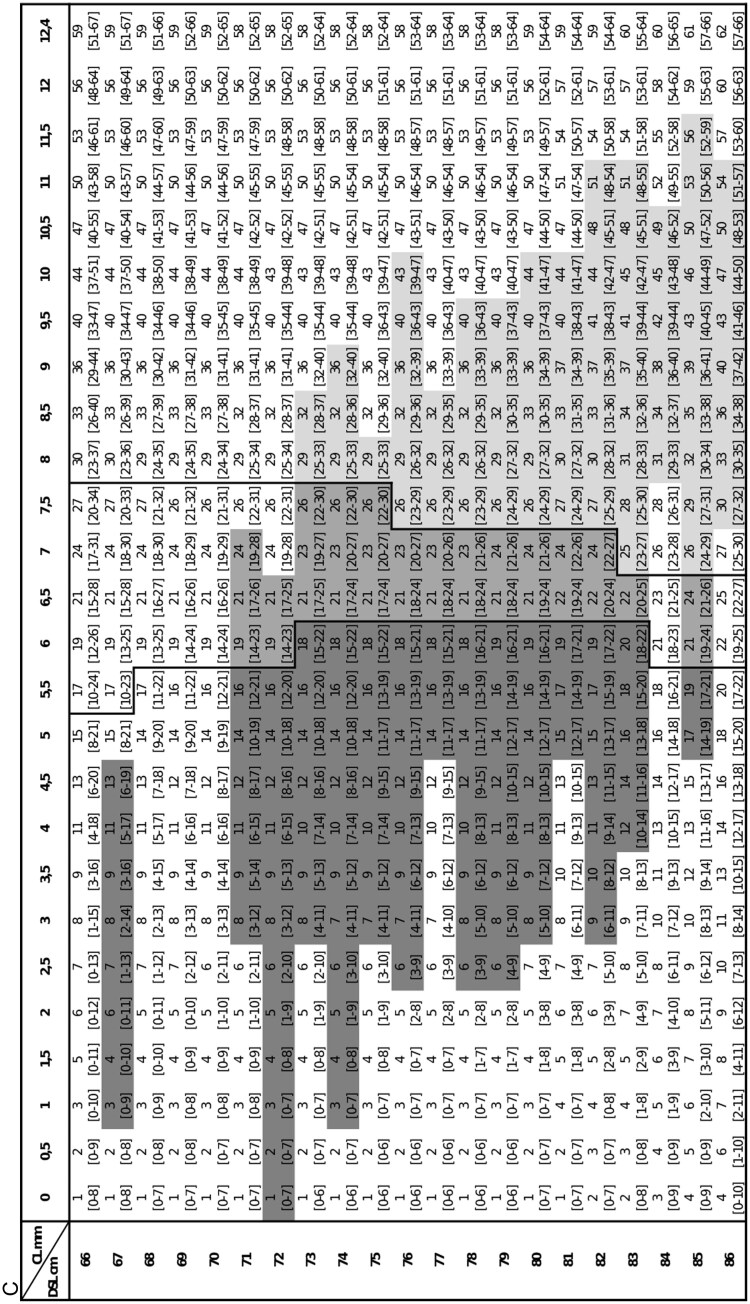

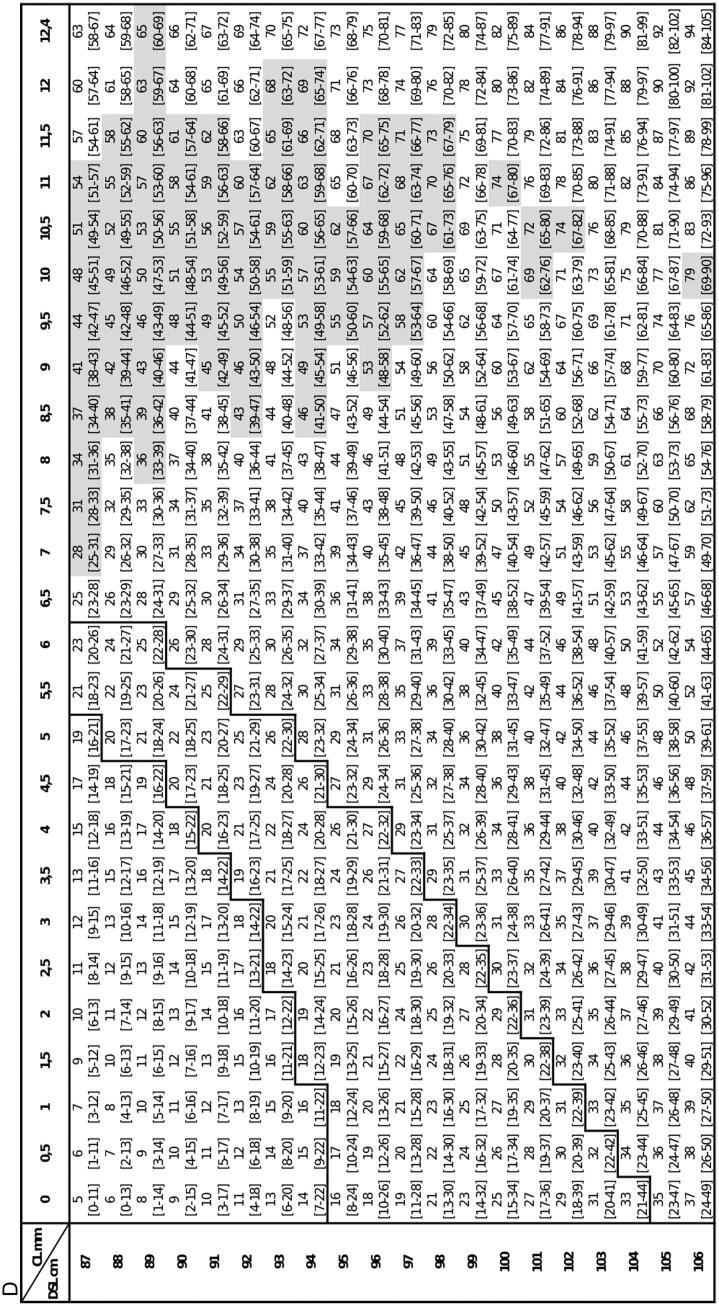
(A, B) Age estimates for male individuals derived from the GAMM, along with their respective 95% confidence intervals (CIs), are provided across various combinations of DSL (cm) and CL (mm). The 95% CIs are indicated within brackets beneath the age estimates. Data points within the observed range are coded using a gray scale, with pups represented by darker gray, weaned individuals by lighter gray, and those with overlapping CIs by medium gray. Distinct delineations between these groups are marked by bold lines. The weaning age is determined as the mean of reported ages in the literature (22 days old). It should be emphasized that not all combinations of DSL and CL are biologically plausible. (C, D) Age estimates for female individuals derived from the GAMM, along with their respective 95% CIs, are provided across various combinations of DSL (cm) and CL (mm). For additional information, please refer to the legend in (A, B).

To translate model results into a practical guideline for estimating the age of a pup when it is found or has been taken in, we created a data set with all possible combinations of DSL and CL. We calculated the age predictions and their standard error (SE) for each combination using the *predict_gamm* function from the *gammit* package (v0.3.2; Clark 2024). We then used SE to calculate the lower and upper limits of the 95% confidence intervals (CIs) for all age predictions. Figure 2A, B, and C, D show the predicted age and the 95% CIs for males and females, respectively.

#### Comparison of growth rate and size at Sealcentre Pieterburen versus wild

The growth rates for DSL and BM were calculated for 45 captive pups. First, we calculated the difference between values measured around the usual time of weaning and values measured at arrival for DSL and BM. Ages of 24 and 31 days old were both used for the calculations because these represent the age at which animals were weaned and measured in 2 different wild populations (Table 1). Growth rate was then calculated by dividing this difference by the number of days that passed between these measurements. We visually confirmed that data were normally distributed and used Welch *t*-tests to compare: (i) captive versus wild growth rates; and (ii) BM values of captive animals at release with those of wild animals at weaning. In all cases, we compared individual data from our captive population with average values of wild populations extracted from the literature, specifically focusing on *P. v. concolor* in Nova Scotia and *P. v. richardii* in British Columbia, since such data were not available for our own population. In the case of DSL growth in Sable Island, Nova Scotia, the literature did not provide the variance and the sample size; therefore, a 1-sample *t*-test was used instead.

**Table 1.** Summary of morphometrics and growth rate of pups in different study areas.

Study	Sable Island, Nova Scotia, Canada (west Atlantic) (1) 1988 to 1996, (2) 1968 to 1973 *Phoca vitulina concolor*	Southern British Columbia, Canada (east Pacific) (1998) *Phoca vitulina richardii*	Sealcentre Pieterburen, The Netherlands (east Atlantic) (2018) *Phoca vitulina vitulina*	
Duration of lactation (days)	23.9 ± 0.26 (175)^(1)(c)^	32 ± 1.5 (28)^(3)^	24 (45)^(a)^	31 (45)^(a)^
Mean BM at weaning (kg)	24.95 ± 0.25 (154)^(1)(c)^	23.6 ± 1.2 (28)^(3)^	10.69 ± 0.16 (45)	12.79 ± 0.23 (45)
BM gain rate (kg/day)	0.6 ± 0.01^(1)(c)^	0.394 ± 0.026 (7)^(3)^	0.1 ± 0.005 (45)	0.16 ± 0.005 (45)
Mean DSL at late lactation^(b)^ (cm)	90^(2)^		78 ± 0.63 (45)	81 ± 0.56 (45)
DSL gain rate (cm/day)	0.58^(2)(c)^	0.33 ± 0.027 (7)^(3)^	0.04 ± 0.05 (45)	0.18 ± 0.03 (45)

Values are given as the mean ± SE when present in the literature. Sample sizes are given between parenthesis. ^(1)^Bowen et al. (2001), ^(2)^Boulva and McLaren (1979), and ^(3)^Cottrell et al. (2002). ^(a)^In captivity, natural processes such as lactation or weaning do not occur; however, we chose 24 and 31 days old to compare the values during rehabilitation with those of wild populations. ^(b)^Late lactation = near weaning. ^(c)^Values for males and females together, calculated from the sex-specific data given in the literature.

#### Effect of BM and BC at arrival on growth curves

We assessed whether BM and BC of a pup on Day 1 of their rehabilitation affect its growth patterns, specifically, the presence of compensatory growth (Metcalfe and Monaghan 2001). Both BM and BC (defined below) were used because each measure exhibits different advantages. BM is commonly used in the literature and allowed us to compare our results with animals raised in the wild, but BC corrects for the size of the animal, potentially making it more biologically relevant. Phocid seals store their energy reserves in the hypodermal blubber layer (Ryg et al. 1988), and consequently the best indices of BC, often represented by the amount of energy stored in tissues (Gales and Renouf 1994), take into account blubber thickness. This was not measured in our study; therefore, we chose the Smirnov Index (SMI) because it strongly correlates with indices that do use blubber thickness (Nilssen et al. 1997). The SMI (Smirnov 1924; cited in several papers including Nilssen et al. 1997), where AG and DSL are measured in cm, is measured as follows:


$$\begin{array}{l} Body\;condition=\frac{AG\times 100}{DSL} \end{array}$$

Consequently, we assessed the state of each animal at arrival using both BM and BC, classifying them into having: (i) poor or good BM; and (ii) poor or good BC. We did this separately for both males and females to factor in possible sexual differences as sex was retained as a predictor in the age model mentioned in the section above. To predict whether pups belonged to the good or poor groups for each measurement, we fitted another GAMM, henceforth known as the state model. Specifically, for this model, we created a new age variable. This age variable has, as a starting value for each pup, its age predicted at arrival obtained from the age model described above. Subsequent age values were simply calculated by adding the number of days that passed since the date of arrival and the dates of following measurements. This was done to make sure that the age values used in our predictions were as realistic as possible. Our state model included the new age variable as a continuous predictor, a random intercept for seal identity, and a random slope for predicted age. We then predicted the (i) BM and (ii) BC for each seal at arrival and compared those predictions with their actual values. If the actual value was lower than the predicted value, the seal was included in the poor group; if the actual value was equal or higher, it was considered part of the good group.

To evaluate if seals in poorer state at arrival compensated by growing faster during the rehabilitation process, we plotted growth curves for: (i) BM, based on the BM groups; and (ii) BC, based on the BC groups. The growth curves were again obtained by fitting GAMMs using a REML approach. All models include both the BM or BC group at arrival of each animal (determined by the state model) and their predicted age (determined by the age model) as fixed effects. We fitted a separate curve with respect to the predicted age for each group, allowing for an interaction between predicted age and the categorical predictor. A random intercept for seal identity and a random slope for predicted age were also included. We plotted the predicted mean and 95% CIs for each group and for the difference between groups across all ages that were included in the data.

## Results

### Examining data and identifying data anomalies

**Fig. 3.**
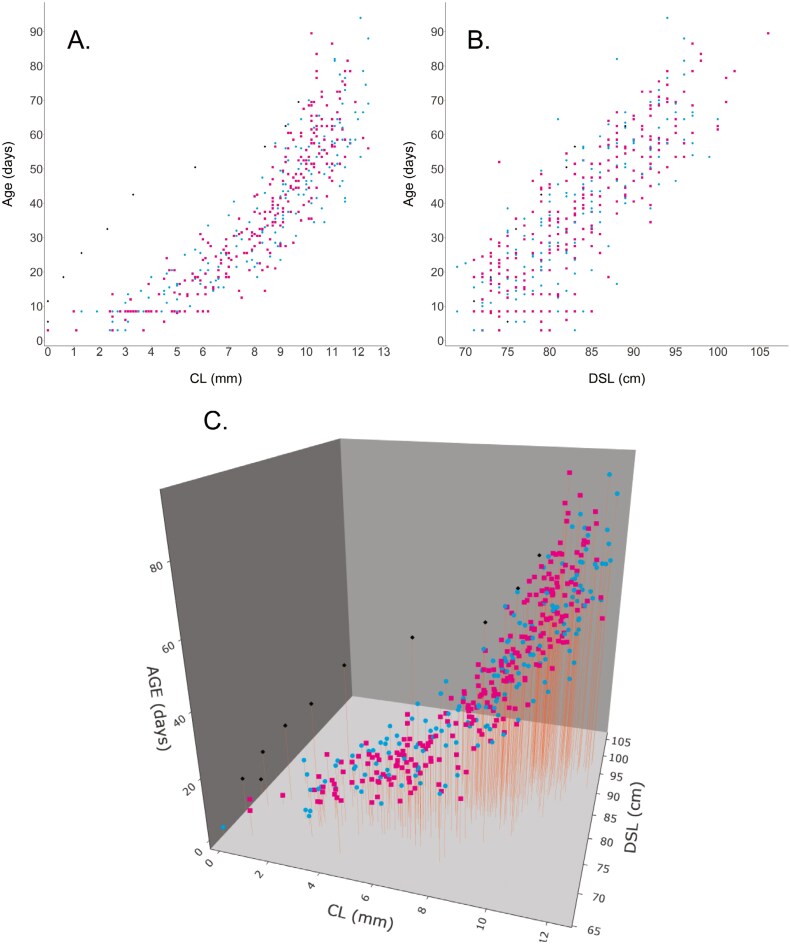
Scatter plot showing the raw data for pups younger than 10 days old upon arrival, utilized in our modeling process. Panel A displays the relationship between CL and age, while Panel B depicts the relationship between DSL and age. In the plots, pink squares signify females, whereas blue dots denote males. Additionally, black diamonds represent an outlier, male 18-081.

Plots of the raw data show that both CL and DSL seem to be important predictors for estimating age in young harbor seals (Fig. 3), justifying our initial decision to include both measures in the minimum model. As depicted in Fig. 3A, the precision of age estimation decreases for higher values of CL, given that it appears to reach an asymptote. Conversely, this pattern is not present for DSL (Fig. 3B), at least within this range. Hence, both CL and DSL together serve as useful predictors. Both trends are jointly visible in Fig. 3C depicting a potential interaction between both. It is important to note the outlier depicted on the left side of Fig. 3A, which corresponds to animal 18-081, whose canines did not erupt until his second week in the Sealcentre. Due to his abnormal CL growth (see Materials and methods section), we decided to exclude this animal from the data set.

### Predicting the age of our study subjects

**Fig. 4.**
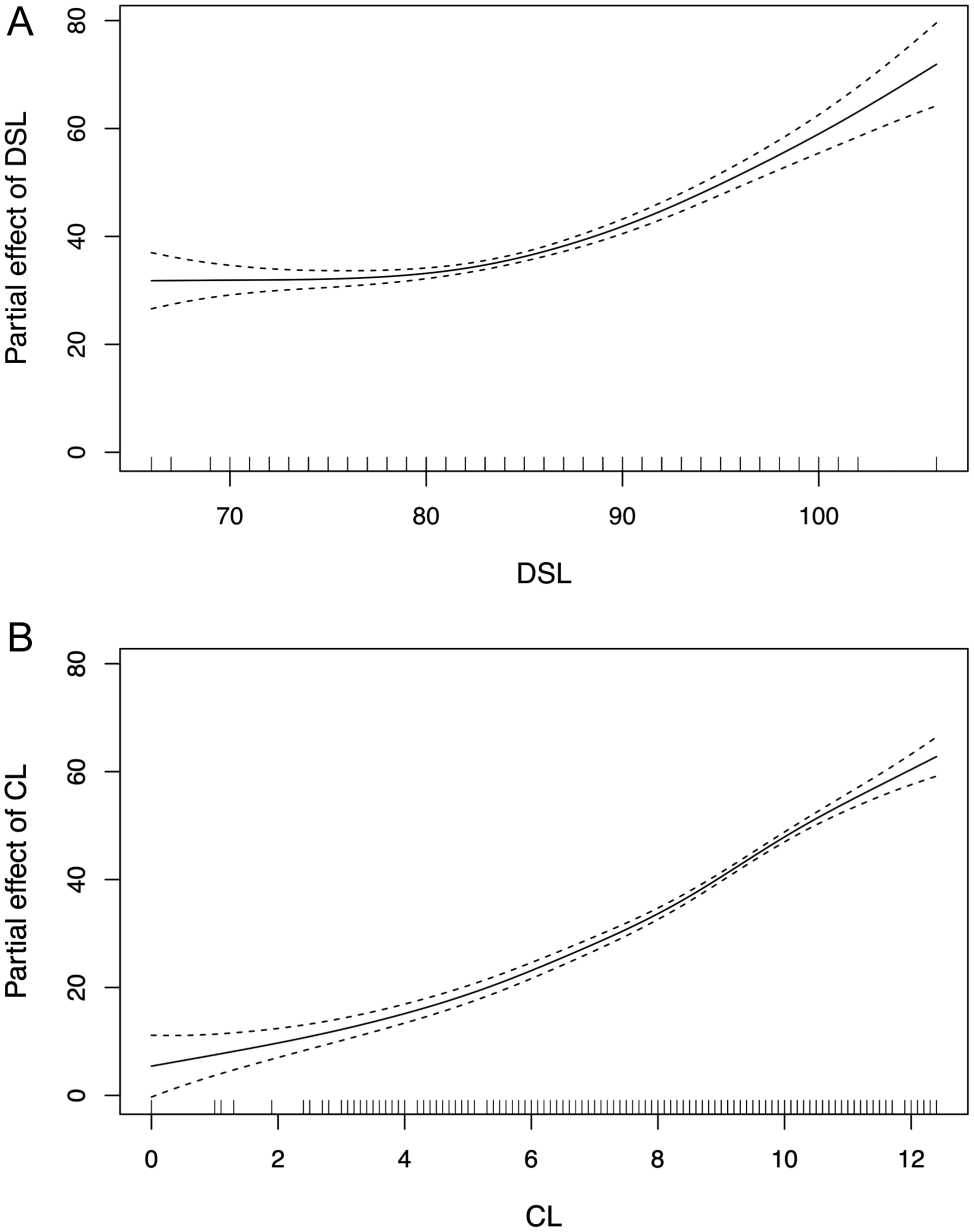
Additive effect of DSL (A) and CL (B) on the estimation of age of young harbor seals.

The stepwise regression analysis revealed that inclusion of interaction terms between DSL and CL, as well as between sex and DSL or CL, did not yield improvements in the predictive capacity of the model. However, inclusion of sex as an individual predictor significantly enhanced performance of the model; thus, it was retained in the final model. Additionally, the incorporation of a random factor characterized by a smooth function for DSL demonstrated a beneficial impact on predictive accuracy of the model. Conversely, the introduction of a similar random factor for CL failed to enhance model performance, leading to its substitution by a random slope. A total of 21 models were fitted during the analysis process. Ultimately, the optimal model, termed the “age model,” included DSL, CL, and sex as fixed effects, in addition to a random factor characterized by a smooth function for DSL and a random slope for CL (Supplementary Data SD6). This model exhibited an adjusted *R*-squared value of 0.952. In the context of GAMMs, a linear predictor typically corresponds to an effective degrees of freedom (edf) value of 1 (Wood 2017). Notably, within our selected age model, both DSL (edf = 4.49) and CL (edf = 4.72) displayed distinct nonlinear associations with age. The effects of the significant fixed effects on age predictions were visualized through plots. Specifically, DSL began to influence predictions once seal length exceeded approximately 80 cm (Fig. 4A)—contrasting with CL, which exerted an effect from the onset of postnatal growth (Fig. 4B).

### Practical guideline to use in the field

Figure 2A–D show the ages estimated and their reliability by the GAMM for a given animal with specific DSL and CL for usage in the field, an important aim of this paper. To facilitate usage, different age classes are plotted in different colors.

### Comparison of growth rate in the Sealcentre Pieterburen versus wild

Table 1 shows that the growth rate at the Sealcentre is significantly lower than that in 2 wild populations in Canada for both BM (Sable Island: *t* = −40.15, df = 153.10, *P* < 0.001; southern British Columbia: *t* = −8.81, df* *= 6.54, *P* < 0.001) and DSL (Sable Island: *t* = −10.12, *P* < 0.001; southern British Columbia: *t* = −3.86, df = 21.30, *P* < 0.001), whether weaning is considered to occur at 24 or 31 days (see Materials and methods section).

In addition, to increase chances of pups surviving after their release back into the wild, pups from the Sealcentre are released with a higher BM than the average weaning BM of that species (29.74 kg in the Sealcentre in 2018 vs. 23.60 to 24.95 kg in the wild). This difference is statistically significant for both wild populations in Canada (Sable Island: *t* = 11.44, df* *= 100.90, *P* < 0.001; southern British Columbia: *t* = 4.93, df* *=* *31.19*, P* < 0.001).

### Effect of BM and BC at arrival on growth

**Fig. 5.**
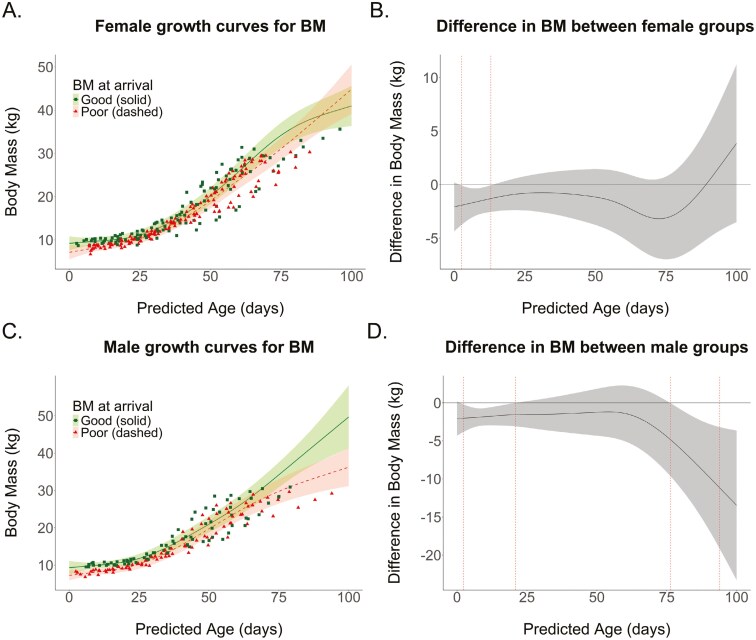
Growth curves for BM depending on BM at arrival for (A) females and (C) males. Squares and triangles depict the raw individual data, while the fitted lines show the model predictions. The plots on the right illustrate the differences in BM between the animals grouped into the poor and good BM categories for (B) females and (D) males. The age ranges marked by red vertical dashed lines indicate periods during which significant differences exist between the 2 groups. Gray areas denote the 95% CI.

Figure 5 shows that females with poor BM at the start seemed to catch up with animals with a good BM when they reached 90 days of age, showing a bit of compensatory growth. Males that arrived at the Sealcentre with a poor BM continued to show lower BM throughout the entire rehabilitation period; differences with the good group seem to become even larger over time. There is a significant difference only at the beginning of the rehabilitation period for females (3 to 13 days old), and at the beginning and toward the end of the rehabilitation period for males (2 to 21 and 76 to 94 days old).

A similar analysis was performed for BC, measured using the Smirnov’s index. Supplementary Data SD7 shows a pattern similar to BM, with animals that start in poor BC remaining in worse condition than those that had a better start. The main difference is that the ranges in which the differences are significant (females: 3 to 31 and 41 to 85 days old; males: 2 to 13 and 43 to 58 days old) are much broader than for BM and that there is no indication for compensatory growth in females.

## Discussion

The purpose of our study was to: (i) find good age predictors in young harbor seals found stranded during the breeding season, placing emphasis on noninvasive techniques that can be used in the field; (ii) compare the growth rates of the animals admitted to the Sealcentre with those in the wild; and (iii) examine whether animals at the Sealcentre show compensatory growth during the rehabilitation process. We determined that DSL and CL could be used to adequately predict age as determined by the condition of their umbilical cord. These measures can be used to determine age beyond 10 days old, when pups typically lose their umbilical cord, making them more useful when evaluating pups or young weaners found stranded in the wild. We found that BM and DSL of animals admitted to the Sealcentre grew at a much lower rate than those weaned by their mothers in the wild. Furthermore, we found no substantial evidence for compensatory growth.

### Predicting the age of our study subjects

The GAMM to predict age (i.e., the age model) proved to be a good choice given how well it fitted the data, suggesting that we could accurately estimate the age of stranded animals in the field. Both DSL and CL together are good predictors for estimating the age of Harbor Seal pups and early weaners, and are also relatively easy to measure in the field without the need to handle the animal for a long period of time. The importance of CL is supported by the model for estimating the age of harbor seal pups created by Skinner (2006) during a field study in Maine in 2004 and 2005. It is important to note that measuring the length of the canine requires professional handling of the seal to get a proper measurement. The other predictors of Skinner’s model were AG and the nursing status of the pup (whether the pup was seen attended by a female or unattended). In our study, AG was not included in the model as it is directly related to nursing status (Skinner 2006) and we had no information about the last time that our subjects were nursed or seen with a female. Furthermore, our model also has limitations related to the nature of our study subjects: they were all admitted into rehabilitation because they were considered orphaned and/or had low-fat scores. Hence, it is highly recommended to replicate this study with animals weaned in the wild, as it would allow us to generalize our age model predictions to wild populations.

### Creating a practical guideline

Reliable age estimation is important as weaners lose BM in the weeks following the end of the lactation period (Muelbert and Bowen 1993), and could thus be easily confused with pups. Figure 2A–D present a useful practical tool that can easily be used in the field, and with a high level of confidence, to identify whether an animal is a pup still being nursed by a female or whether it is already weaned. These tables will facilitate the decision-making process of rehabilitation centers concerning which animals need help (i.e., pups) and which are independent enough to survive on their own (i.e., young weaners). Nevertheless, it could be that the animal is sick and may need help, independent of its age. It is important to emphasize that the tables are meant to be used by a minimum of 2 people: 1 restraining the animal and the other measuring it. As such, they must be trained in the proper handling of seals to ensure accurate measurements. It is worth mentioning that, given that the data are sourced from rehabilitated animals and comparisons with wild counterparts from distinct populations indicate differences in growth rates, these tables may prove more beneficial when identifying potential orphans rather than assessing the entire wild population.

### Comparison of growth rate and size at Sealcentre Pieterburen versus wild

Our results show that overall BM growth rate at the Sealcentre Pieterburen (0.1 to 0.16 kg/day) was significantly lower than that for wild-raised pups (0.394 to 0.600 kg/day). This discrepancy is in line with the findings of MacRae et al. (2011) on Harbor Seal pups recovered along the British Columbia coastline and admitted into rehabilitation (0.043 to 0.123 kg/day). It is remarkable that our study findings agree with those of MacRae et al. (2011) despite different definitions of when we consider an animal to be weaned in rehabilitation. They considered animals to be weaners once they were fed whole fish in all feedings (20 to 30 days old) and we based our definition on the average weaning age for seals in the area (24 days old), independently of their type of diet. Our results are also supported by the findings of Richmond et al. (2010) who stated that rehabilitated Harbor Seal pups grew in terms of BM at a much slower rate than animals in the wild. No literature was found for pups and young weaners raised in captivity regarding the growth rate of DSL.

### Effect of BM and BC at arrival on growth curves

We did not find a clear indication for compensatory growth for either BM or BC; in general, animals that started rehabilitation with a poor BM or BC had a lower BM or BC throughout their stay, with the exception of the BM of females over the age of 90 days old. However, this fact should be considered carefully as just 1 single female individual reached 90 days of age at the Sealcentre. Because this individual belonged to the good BM group, its data have highly affected the shape of the curve. We do not know how the curve would have looked for an animal of the poor BM group at 90 days of age. There may be several possible explanations for not being able to clearly identify compensatory growth. Firstly, it could be that the differences between poor and good groups are so small that we are not able to see compensatory growth, probably because all of our subjects were considered underconditioned at arrival. This possibility aligns with the statement from Hornick et al. (2000), that young, food-restricted ungulates often fail to express compensatory growth. Secondly, it may be that young harbor seals do not show compensatory growth due to compensatory strategies of the mother (Kovacs and Lavigne 1986). Furthermore, lack of evidence for compensatory growth raises the question whether the feeding protocol in the Sealcentre allows for compensatory growth or whether the animals do not have the physiological capacity for it—a question for further study. It is important to note that the mass of a mammal at weaning reflects the extent to which it is prepared to cope with its adult niche (Millar 1977). For example, weaning mass is highly correlated with 1-year survival in several pinniped species: gray seals, *Halichoerus grypus* (Hall et al. 2001); southern elephant seals, *Mirounga leonina* (McMahon et al. 2000); northern fur seals, *Calorhinus ursinus* (Baker and Fowler 1992). Harding et al. (2005) calculated that harbor seal pups must reach 25 kg before fall to increase their chances of surviving the winter. Kovacs and Lavigne (1986) found that female seals nurse their pups for longer if needed to ensure that they reach a weaning weight that improves their chances of survival. Hence, it seems that females have found an alternative solution to make up for the size of smaller-born pups. Interestingly, rehabilitation systems follow similar procedures to give animals a better chance of survival: their stay lasts until they have reached a good BM or BC that sustains them for longer while they learn how to forage (MacRae et al. 2011). Finally, it may be that they do show compensatory growth, though later in time. Unfortunately, our animals were all very young (i.e., pups or early weaners); hence, we could not test for compensatory growth at older life stages. Given that seal centers often release animals once they have reached a sufficient BM (among other criteria), in fact mimicking the maternal strategy mentioned above, it is important to tag animals at release and monitor their survival rate and perhaps their reproduction because the rehabilitation protocols may affect seal population dynamics.

The growth curves and the above-cited literature suggest that animals starting with a low BM or BC may also be at disadvantage in terms of winter survival. Although speculative, one other possible consequence of being born with poor BM or BC is that mating performance of males may be affected. Male harbor seals vocalize underwater to attract females (Hanggi and Schusterman 1994) and establish territories along female-traffic corridors (Hayes et al. 2004). As the body size of harbor seals is directly proportional to the size of their vocal tract (de Reus et al. 2022), their ability to defend underwater territories/corridors and gain access to females may be affected, given that size differences influence the call frequencies (pitch) they can produce.

The growth curve plots show some differences between females and males. Pups that arrived with poor BM did not show compensatory growth, except perhaps for females after 80 days old. However, the presence or absence of compensatory growth for either sex at that age may have to do with our model fit, which is greatly influenced by the limited number of older individuals (Fig. 4A and C). Hence, the shape of the curve between 80 and 100 days old may not be representative of the larger population. One possibility for ruling out the presence of compensatory growth at older ages might involve extending the duration of animal rehabilitation. However, this contradicts the principle that animals should not be kept in captivity beyond what is essential. It is interesting to note the shape of the curve for males in good BC at the start of rehabilitation, which unexpectedly decreased over the first 20 days. This result may, however, be an artifact of our data due to the large variability in the BC of young males belonging to the good group, likely caused by the small sample size (Supplementary Data SD7). No such artifacts are observed for female BC or for males in poor BC, likely due to the higher number of individuals in those groups (Supplementary Data SD7).

In conclusion, this study provides an objective guide for assessing the age of young Harbor Seal pups suspected of being prematurely abandoned by their mothers in the field. This guide is useful not only for rehabilitation centers but also for studies on Harbor Seal behavior, biology, and ecology. Finally, the Harbor Seal population in the Wadden Sea is abundant and healthy, raising the question of whether we need to continue rehabilitating them. Being able to accurately assess the age of an animal may therefore reduce the number of animals that are unnecessarily brought into rehabilitation.

## Supplementary data

Supplementary data are available at *Journal of Mammalogy* online.

**Supplementary Data SD1.** Location of the finding spots in the North of the Netherlands for the 45 Harbor Seal (*Phoca vitulina vitulina*) pups that were included in the study.

**Supplementary Data SD2.** Fat scores of harbor seals (*Phoca vitulina vitulina*) derived from the body condition score system outlined by Merck (2013).

**Supplementary Data SD3.** Estimated age of Harbor Seal (*Phoca vitulina vitulina*) pups based on the state of the umbilical cord.

**Supplementary Data SD4.** Curve shapes of the relationship of age with DSL of the 45 Harbor Seal (*Phoca vitulina vitulina*) pups included in the study.

**Supplementary Data SD5.** Curve shapes of the relationship of age with canine length (CL) of the 45 Harbor Seal (*Phoca vitulina vitulina*) pups included in the study.

**Supplementary Data SD6.** Most comprehensive (m1) and optimal GAMMs from the stepwise backwards elimination regression process.

**Supplementary Data SD7.** Growth curves for body condition (BC) depending on BC at arrival, for (A) females and (C) males. Squares and triangles depict the raw individual data, while the fitted lines show the model predictions. The plots on the right illustrate the differences in BC between the animals grouped in the poor and good BC categories, for (B) females and (D) males. The age ranges marked by red vertical dashed lines indicate periods during which significant differences exist between the 2 groups. Gray areas denote the 95% CI.

gyae128_suppl_Supplementary_Data_SD1

gyae128_suppl_Supplementary_Data_SD2

gyae128_suppl_Supplementary_Data_SD3

gyae128_suppl_Supplementary_Data_SD4

gyae128_suppl_Supplementary_Data_SD5

gyae128_suppl_Supplementary_Data_SD6

gyae128_suppl_Supplementary_Data_SD7

## Data Availability

Data are available at https://doi.org/10.34894/RXDQSN.
