# Supplementary figures and images for: Age estimation and growth patterns in young harbor seals (Phoca vitulina vitulina) during rehabilitation
Source: J Mammal. 2024 Dec 10;106(2):491–504. doi: 10.1093/jmammal/gyae128 (PMC11933287; doi:10.1093/jmammal/gyae128)

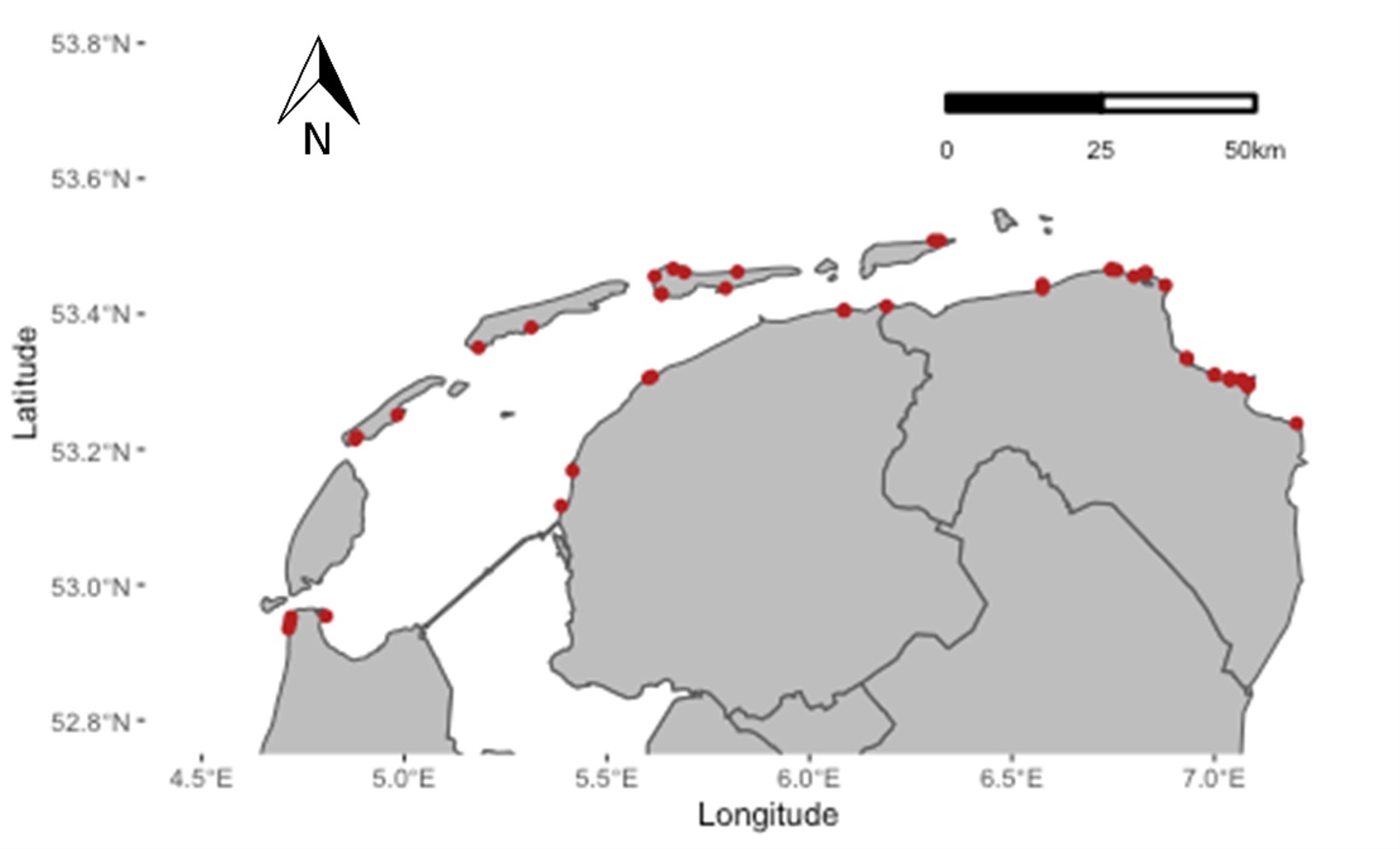

Supplement: gyae128_suppl_Supplementary_Data_SD1 [file gyae128_suppl_supplementary_data_sd1.jpeg]

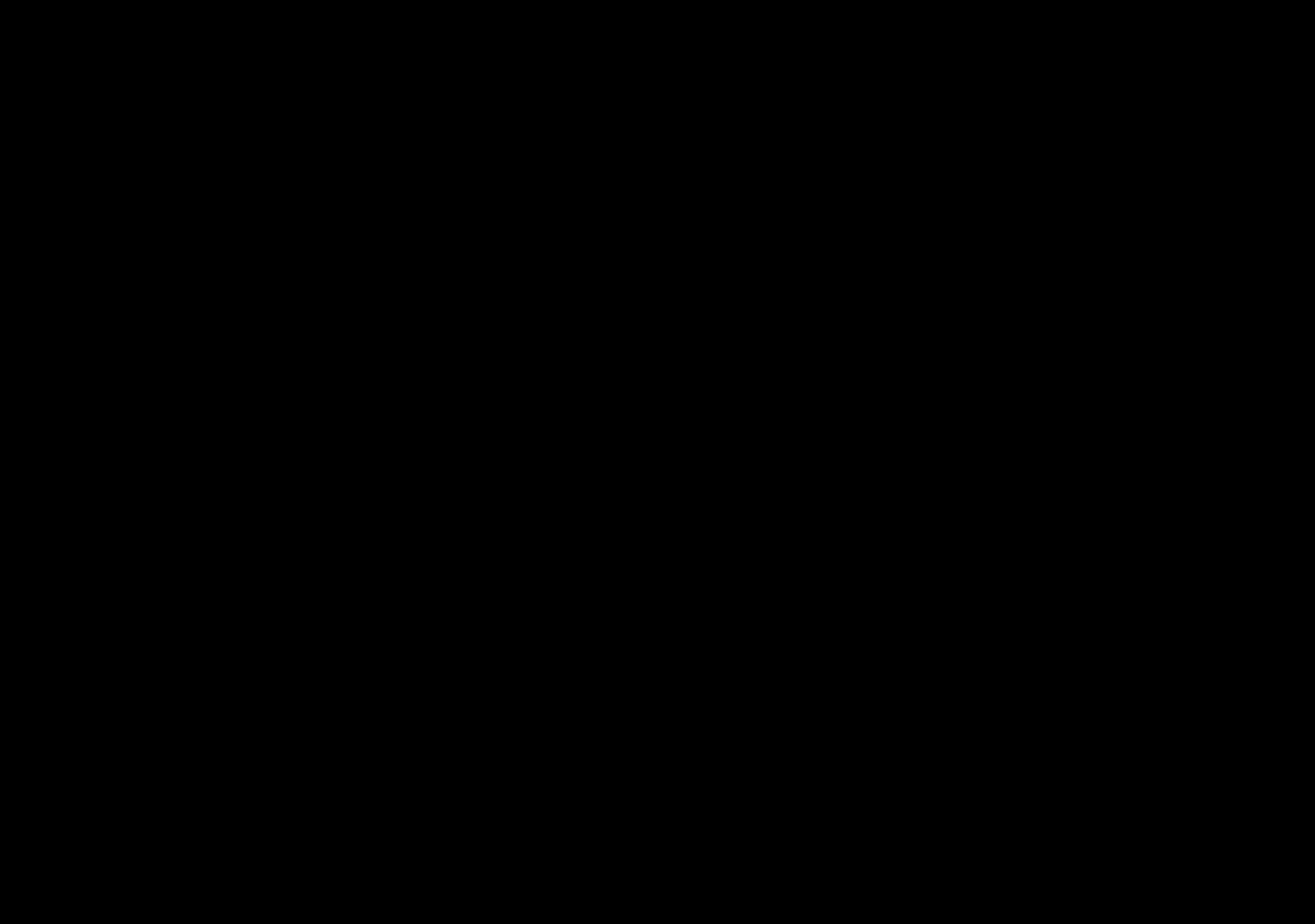

Supplement: gyae128_suppl_Supplementary_Data_SD2 [file gyae128_suppl_supplementary_data_sd2.jpeg]

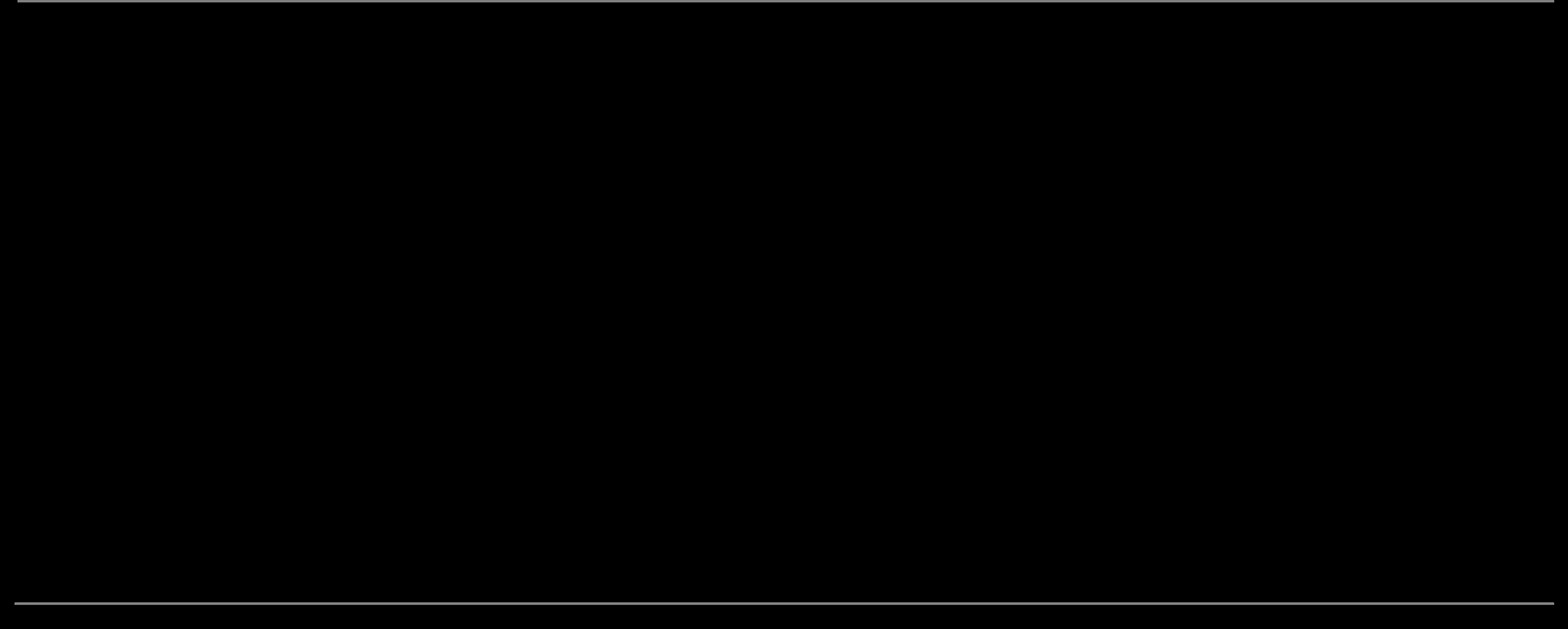

Supplement: gyae128_suppl_Supplementary_Data_SD3 [file gyae128_suppl_supplementary_data_sd3.jpeg]

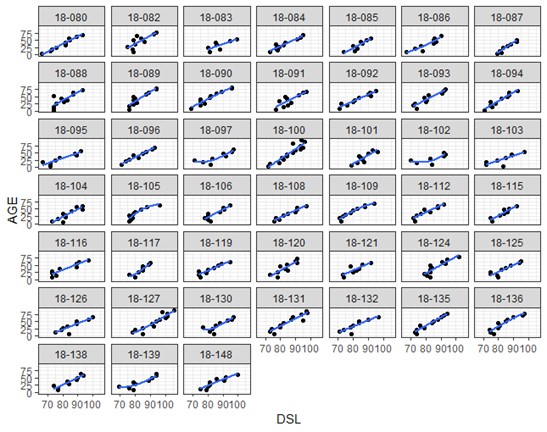

Supplement: gyae128_suppl_Supplementary_Data_SD4 [file gyae128_suppl_supplementary_data_sd4.jpeg]

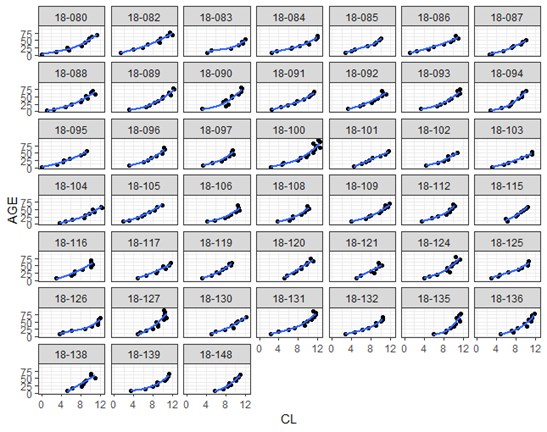

Supplement: gyae128_suppl_Supplementary_Data_SD5 [file gyae128_suppl_supplementary_data_sd5.jpeg]

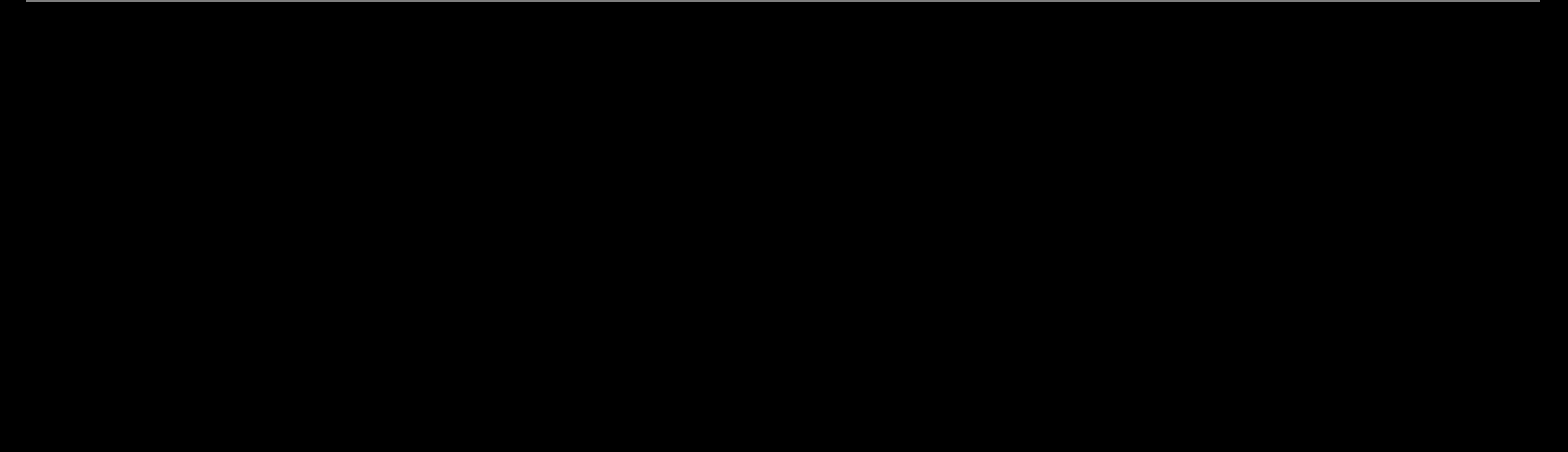

Supplement: gyae128_suppl_Supplementary_Data_SD6 [file gyae128_suppl_supplementary_data_sd6.jpeg]
